# Selected properties of Al*_x_*Zn*_y_*O thin films prepared by reactive pulsed magnetron sputtering using a two-element Zn/Al target

**DOI:** 10.3762/bjnano.13.29

**Published:** 2022-03-31

**Authors:** Witold Posadowski, Artur Wiatrowski, Jarosław Domaradzki, Michał Mazur

**Affiliations:** 1 Wroclaw University of Science and Technology, Faculty of Electronics, Photonics and Microsystems, Janiszewskiego 11/17, 50-372 Wrocław, Poland

**Keywords:** aluminium zinc oxide, magnetron sputtering, thin film, transparent conducting oxide, transparent electronics

## Abstract

Al*_x_*Zn*_y_*O thin films were obtained by reactive pulsed magnetron sputtering. A two-element Zn/Al planar target was used as source material prepared in the form of a Zn disc (100 mm diameter) with Al rings pressed into its surface. The sputtering processes were carried out in a mixture of argon and oxygen. The films were deposited with a discharge power of *P*_E_ = 400 W, which corresponded to a power density on the target surface of approximately 5 W/cm^2^. The films were deposited on glass strip substrates, placed symmetrically over the target, making it possible to obtain films with different composition and thickness. The film sheet resistance was measured as a function of the distance from the target axis on both sides (front and back) of the substrate. The lowest measured resistivity was about 4 × 10^−3^ Ω·cm. Additionally, optical properties, surface topography, and elemental composition were determined in selected areas of the substrate.

## Introduction

Aluminium-doped zinc oxide (AZO) is a potential alternative to indium tin oxide (ITO) for transparent conducting oxide (TCO) electrodes in transparent electronic and photovoltaic devices (e.g., touch screens, light emitting diodes, optoelectronics, and photovoltaic organic devices) [1–5]. AZO has good long-term stable electrical and optical parameters, including high electrical conductivity and high optical transmission in the visible range. For the purpose of manufacturing transparent electrodes, the TCO films should have relatively low resistivity, ρ_TCO_ ≤ 10^−3^ Ω·cm, and optical transmittance, preferably over 80%, for films with thicknesses not exceeding 200 nm. In addition, parameters such as high resistance to mechanical exposure and good adhesion to the substrate are strongly desired. Magnetron sputtering is one of the most widely used methods for obtaining thin films with different properties for different purposes and is one of the most commonly used methods for industrial production. However, the manufacturing of AZO thin films is a challenge and requires many preliminary experiments to find optimum conditions for the deposition of thin films with desired properties.

For the deposition of AZO thin films by magnetron sputtering, the most commonly used targets are made of sintered zinc oxide powders and aluminum oxide (ZnO/Al_2_O_3_). The optimal percentage of aluminum oxide in such targets is approximately 2% [6–18]. However, in research in which the objective was to obtain films with minimum resistivity, the alumina content was typically varied in the range of 1–6% [8–9,12].

AZO films have also been obtained so far by reactive sputtering of Zn/Al metal targets [8,10,12–14,19–21] using an argon/oxygen atmosphere. There are also examples where such films were deposited by co-sputtering of two independently powered ZnO and Al targets [10,20–21]. This method of sputtering allowed the composition of the deposited films to be changed by changing the electrical parameters of the two power supplies. Usually, in the abovementioned sputtering processes, the reported power density at the target was of the order of a few watts per square centimeter. Additionally, the substrates were intentionally heated to 500 °C. The lowest measured resistivity of the investigated thin films was in the range from 1 × 10^−4^ to 1 × 10^−3^ Ω·cm. Furthermore, the prepared films were characterized by a light transmittance greater than 80%. Post-process annealing was also found to result in reduced resistivity and increased optical transmission [10,20–21].

In the magnetron sputtering process, it is possible to influence the properties of deposited films by varying the composition of the sputtering gas atmosphere, the total gas pressure, substrate temperature and bias, the target–substrate distance, the target power density, and the configuration of the magnetron magnetic field (balanced/unbalanced). The geometry of the deposition apparatus, that is, how the substrates are arranged inside the vacuum chamber with respect to the sputtering source, also plays an important role. Among other things, it is possible to arrange the deposition process of films using the so-called on-axis (substrates placed directly face to the sputtered source material) or off-axis (substrates placed outside or angled to the target) geometry. By changing the geometry from on-axis to off-axis it is possible to change the intensity of interaction of charged particles (mainly electrons that escape from the magnetic trap directly over pole pieces) with the growing film. Using on-axis geometry, it was found that secondary electrons emitted from the target and negative oxygen ions influence the texture of the growing AZO film and decrease its conductivity [17]. It was also proven that electrically conductive and transparent AZO films could be deposited using the off-axis geometry [11,15,17]. With respect to the axis of magnetron, the substrates were placed at a distance greater than the radius of the target, that is, outside the erosion zone and outside the external pole piece of the magnetron. In such a configuration, substrates were placed in the region where both electrons emitted from the target and the negative ions of oxygen were not present.

Summarizing the review of literature resources, it can be pointed out that regardless of the type of magnetron powering (dc, rf, or dc with rf support) or the type of target used (sintered powders or metallic), a target power density of about 4 W/cm^2^ is typical for the deposition of AZO films with comparable properties (resistivity and optical transmission).

The objective of the research reported here was to present the process of deposition of Al*_x_*Zn*_y_*O films using an original (self-invented) two-element Zn/Al planar target prepared in the form of a Zn disc (100 mm of diameter) with Al rings pressed into its surface. To the best of our knowledge, the results of investigations on Al*_x_*Zn*_y_*O films prepared in this way have not been presented in the literature so far. In comparison to sintered or alloy targets, our approach of preparing the target is simple, and it is easy to introduce modifications of the target composition (i.e., the amount of added Al). Several preliminary sputtering processes were performed to determine the appropriate amount of Al inserts in the Zn disc target and to determine the sputtering conditions under which conducting and transparent films could be deposited.

The goal presented in this paper was to determine the influence of substrate placement (on- or off-axis geometry) and to find favorable conditions for the preparation of the Al*_x_*Zn*_y_*O films, which comprises requirements for the manufacturing of TCO films.

## Experimental

**Figure 1:**
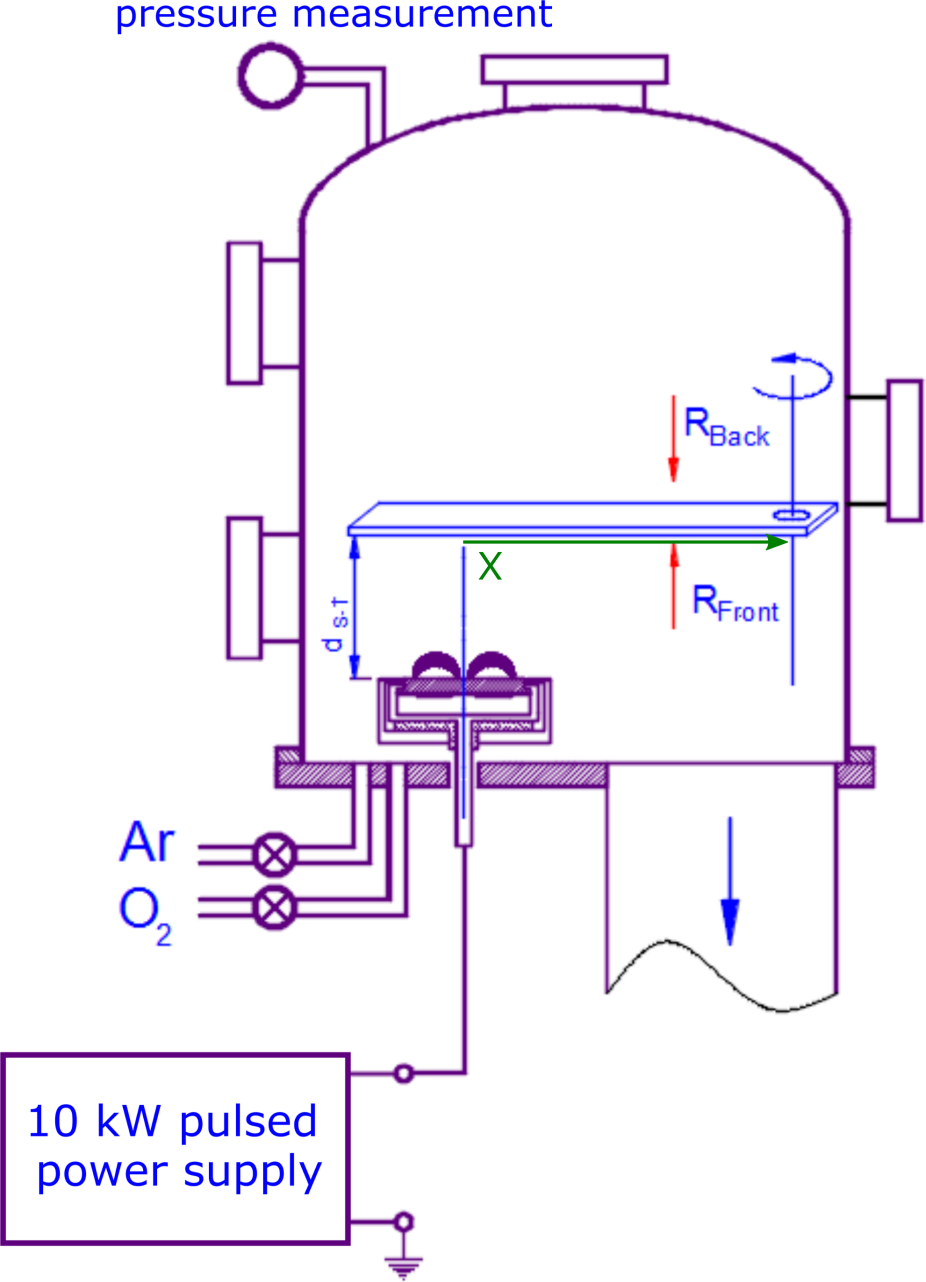
Schematic diagram of a sputtering system for the deposition of Al*_x_*Zn*_y_*O films.

The sputtering system used for the deposition of Al*_x_*Zn*_y_*O films consisted of a custom-made circular magnetron with an originally self-invented two-element Zn/Al target and a pulsed (100 kHz) Dora Power Systems power supply, a MSS-10kW type. Films were deposited using a vacuum stand equipped with a diffusion pump (2000 L/s) and a rotary pump (30 m^3^/h). The ultimate pressure in the working chamber was about 3 × 10^−5^ mbar. A circular magnetron sputtering source, suitable for sputtering targets with a diameter of 100 mm, was installed in the vacuum chamber (Figure 1). The magnetron uses a standard circular magnetic system based on NdFeB magnets (internal N pole and external S pole) that ensures the operation in the balanced mode.

**Figure 2:**
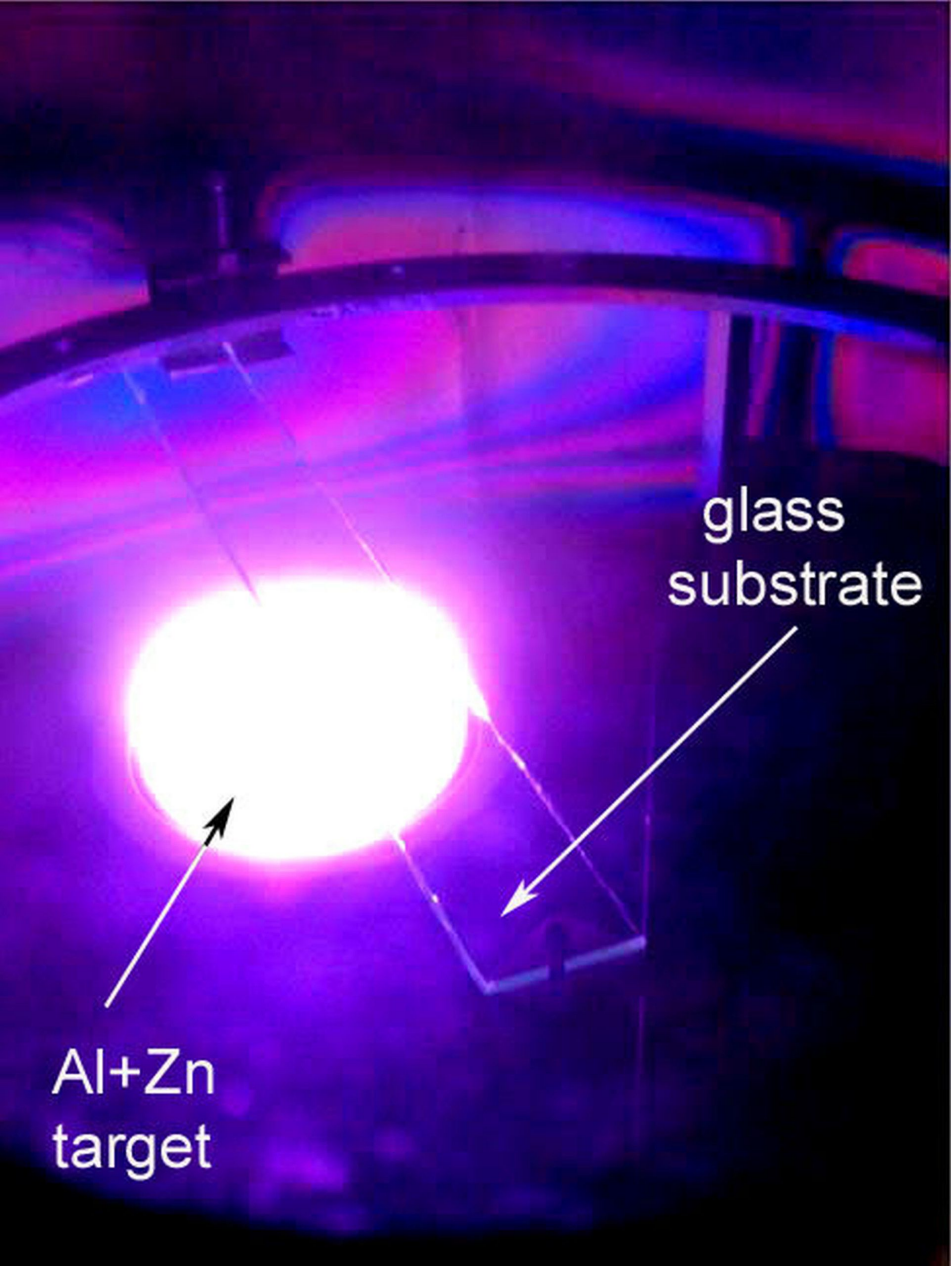
View of the working magnetron and glass strip substrate positioned over the Zn/Al target.

A mixture of working and reactive gases, namely argon and oxygen, with a ratio of 70:30, was introduced into the vacuum chamber through a set of needle valves (Figure 1). The total pressure during reactive sputtering processes was established at about *p*_Ar+O2_ = 3 × 10^−3^ mbar. The power supplying the target (the discharge power) was equal to 400 W. The power supply is the medium frequency pulsed power supply, inducing a pulsed magnetron discharge. The output current pulses are sinusoidal with a duration of 10 µs and a stabilized amplitude of 16 A. During each current pulse, with changes in the magnetron discharge impedance, the supply output voltage varies to keep the sinusoidal shape of the magnetron current. Since the power supply operates in a pulsed manner (100 kHz), the average output power is controlled via pulse quantity modulation with a gating period of about 1 ms. The target–substrate distance was *d*_S–T_ = 100 mm and the thickness of the Zn disc was *d*_ZnAl_ = 9 mm. The films were deposited on glass strips (3 × 20 × 350) mm^3^ placed parallel to the target surface (Figure 1 and Figure 2). Such a configuration allowed for the deposition of the films in two types of geometry: on-axis, where the part of the substrate was directly above the target, and off-axis, where the part of the substrate was outside the target area.

**Figure 3:**
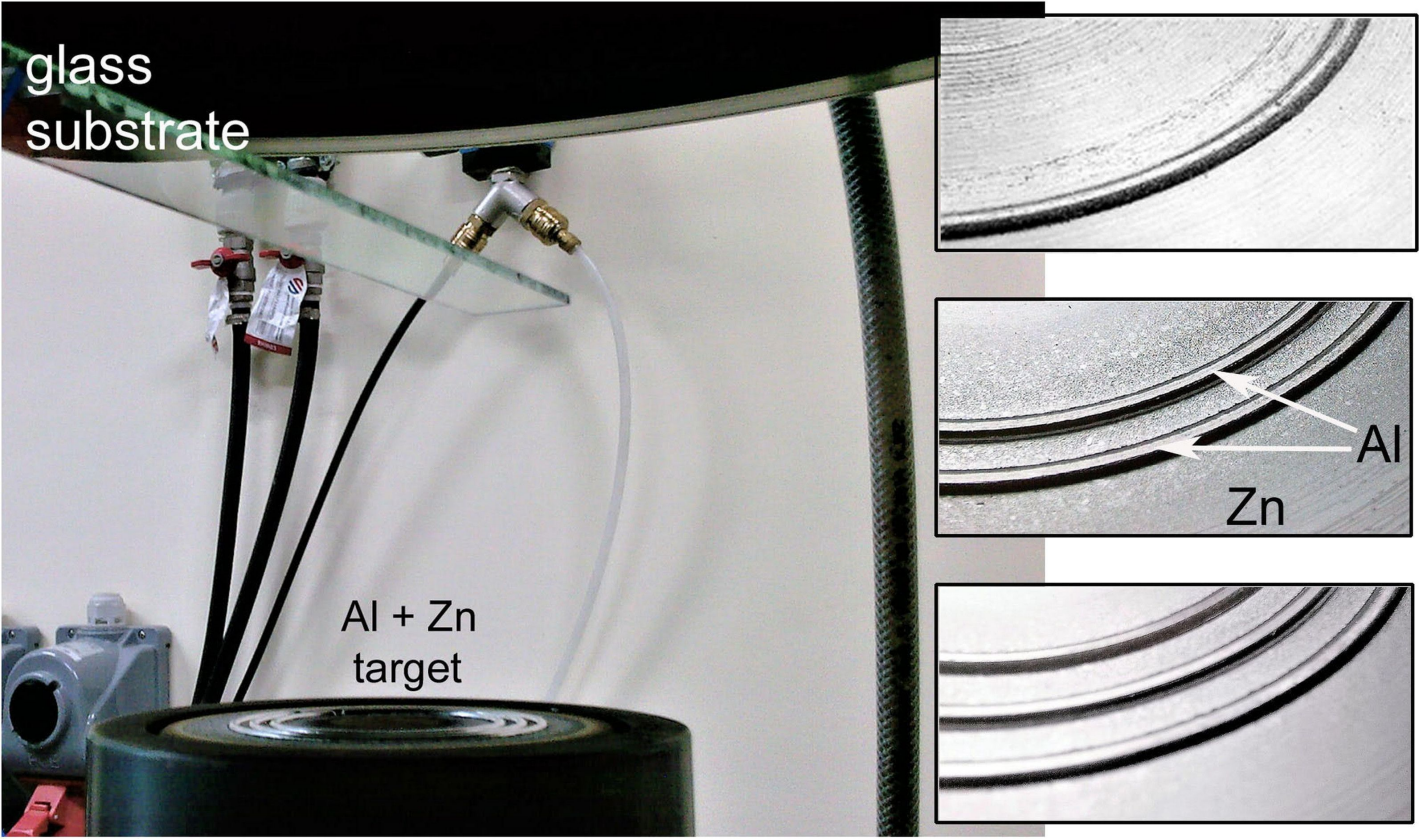
Magnetron source with Zn/Al target and images showing tested arrangements of Al inserts.

The two-element Zn/Al target was prepared by pressing circular Al rings (Al wire with diameter of 2 mm and purity of 99.999%) inside milled grooves of a 100 mm diameter Zn disc surface (Figure 3). The placement and number of Al rings were estimated taking into account the sputtering yield of Zn and Al (at 500 eV of the Ar ion energy *Y*_Zn_ = 5 and *Y*_Al_ = 0.9 [22]) and the width of the race track (25 mm) of our magnetron source. Finally, three Al rings with diameters of 55, 60 and 65 mm were used, which was experimentally specified on the basis of the target power density and the ratio of the Ar/O_2_ mixture to ensure the stability of the composition of the deposited films with progressive erosion of the target material. The detailed parameters of the deposition process are summarized in Table 1.

**Table 1:** Parameters of the deposition process.

base pressure (mbar)	3 × 10^−5^
working pressure (mbar)	3 × 10^−3^
Ar/O_2_ ratio (%)	70:30
magnetron discharge power (W)	400
deposition time (s)	900
target-substrate distance (mm)	100
target diameter (mm)	100
target thickness (mm)	9

The sheet resistance of the deposited thin films was determined using a standard four-point probe head (Jandel Engineering Ltd.) and a source-measure unit (Keithley 2611A type). The measuring head was equipped with four tungsten carbide needles, which were arranged in line with a needle-to-needle distance of 1.00 mm. The measurement was carried out such that the line connecting the four needles was perpendicular to the longest dimension (350 mm) of the glass stripe.

The optical properties were evaluated on the basis of the transmission spectra. The characteristics were acquired using an Ocean Optics spectrophotometer (QE65000 type) and a coupled halogen–deuterium lamp in the wavelength range of 300 to 1000 nm. Transmitted light was collected using an integrating sphere with a perpendicular incidence of the light beam on the sample for nonpolarized light. Analysis of optical properties was performed using the WTheiss Hard- and Software Scout software, ver. 4.17.

X-ray diffraction in the grazing incidence mode (GIXRD) was used to assess the structural properties of the deposited thin films. For this purpose, an Empyrean PANalytical X-ray diffractometer equipped with a PIXel3D detector and a Cu Kα X-ray source with a wavelength of 1.5406 Å was used. The diffraction patterns were collected with a step equal to 0.05°, a time per step of 5 s in the 2θ range of 30° to 80° and the incidence angle of the Cu Kα radiation was constant and equal to 3° relative to the sample surface. The morphology of the surface and cross section of the deposited thin films was investigated using a FEI Helios NanoLab 600i scanning electron microscope coupled with an energy-dispersive X-ray spectrometer (EDS) to determine the amount of Al and Zn in the deposited films (without taking the oxygen signal into consideration).

## Results

**Figure 4:**
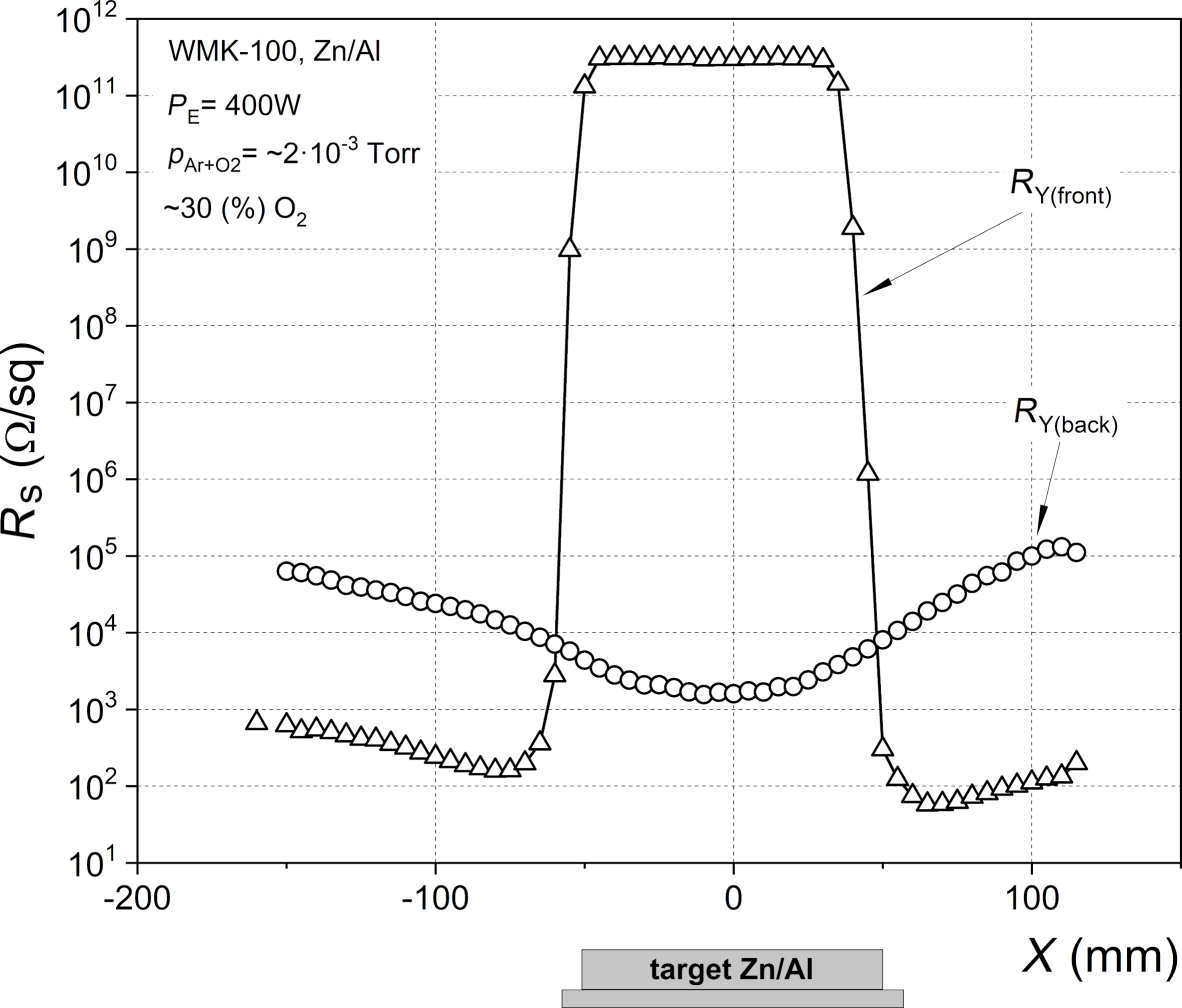
Surface resistance measured for the thin film deposited on both sides of the glass substrate as a function of the distance *X* from the Zn/Al target axis. The line is to guide the eye.

The diagram in Figure 4 shows the surface resistance of the film as a function of the position on the substrate with respect to the axis of the target, *X*. Directly above the target, the film on the front surface of the substrate (Figure 4, front) had a resistance of 3 × 10^11^ Ω/sq. The lowest sheet resistance, approximately 100 Ω/sq, was measured in the area close to the edge of the target. A tremendous change in the film sheet resistance, of about nine orders of magnitude, appears in the area close to the edge of the target.

The opposite result was observed for the surface resistance measurement performed on the back side of the glass substrate (Figure 4, *R*_Y(back)_). The surface resistance measured at the place corresponding to the target center was seven to eight orders lower than on the front side. The lowest resistance, about 10^3^ Ω/sq, was measured directly near the target axis. Resistance measurements performed in places corresponding to the areas outside the target resulted in higher values; however, the difference to the resistance values measured at the center area of the substrate back side was not higher than two orders.

**Figure 5:**
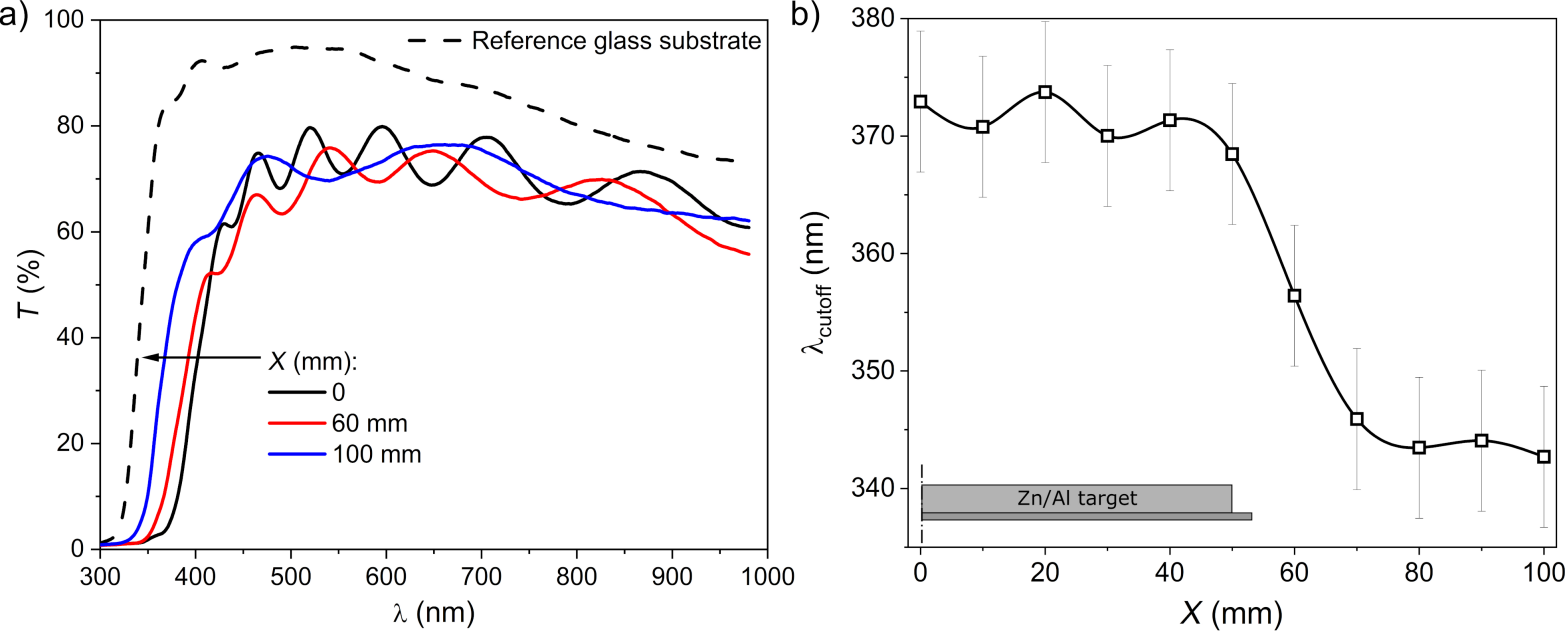
Results of investigations on optical transmission: (a) transmission (*T*) spectra and (b) dependence of the cut-off wavelength on the distance *X* from the Zn/Al target axis. The line in (b) is to guide the eye.

Another parameter important for the practical use of the TCO thin films for the purpose of transparent electronics is the transparency in the visible part of the optical spectrum. Figure 5a shows the light transmission (*T*) characteristics of the prepared thin films measured in the spectral range from 300 to 1000 nm as a function of the distance from the target axis (due to the symmetry, the figure shows results only for one side of the target; compare Figure 4). It is worth noting that the presented transmission characteristics were determined for the entire structure, that is, including the films on both the front and rear sides of the glass substrate, and the glass substrate itself. For comparison, the thin film spectra are presented together with the *T* characteristic of a bare glass substrate. As can be seen, all films, measured at different places of the substrate as a function of *X*, were well transparent, with an average transmission of about 70%. As one moves away from the target axis, a change in the distribution of the interference minima and maxima can also be noticed. The highest concentration of minima and maxima interferences occurs directly in the vicinity of the target axis. As one moves away from the target axis, the minima and maxima move apart. The observed changes suggest that the film thickness decreased with *X*.

To better visualize the effect of the substrate placement on the optical properties of the deposited films, in Figure 5b, the fundamental absorption edge (the cut-off wavelength, λ_cut-off_) as a function of *X* is presented. In the area directly above the target (*X* = 0–50 mm), the position of the optical absorption edge of thin films is quite similar (Figure 5b). Moving away from the axis beyond the area above the target (*X* = 50–100 mm), the optical absorption edge of the spectra shifts in the direction towards shorter wavelengths, from about 370 to 342 nm (Figure 5b).

**Figure 6:**
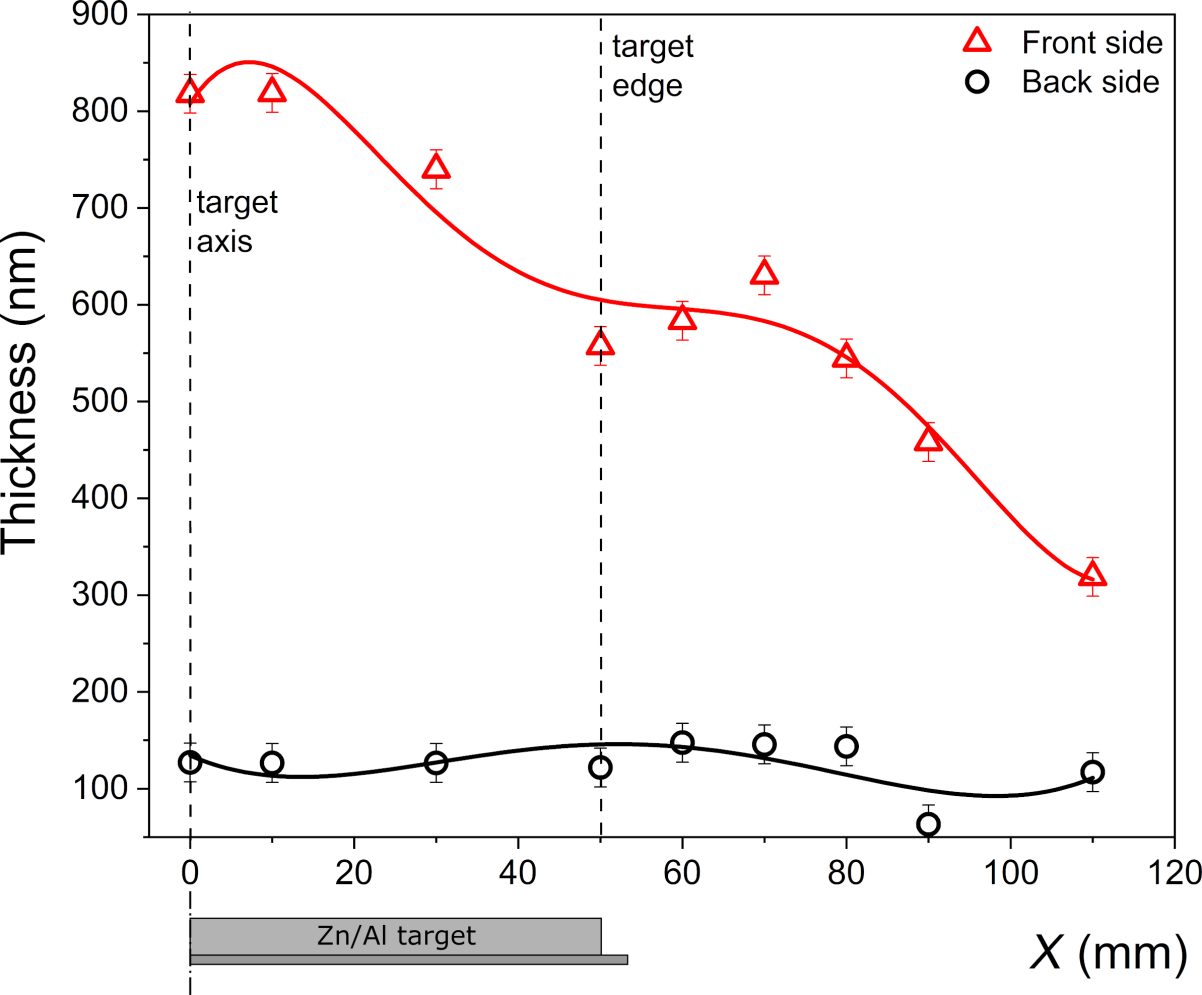
Dependence of thickness of thin films deposited on the front and back sides of the substrate as a function of the distance *X* from the Zn/Al target axis. The lines are to guide the eye.

The measured light transmission characteristics were further used to determine the thickness and the optical bandgap energy of the prepared films. For the analysis, the reverse synthesis method was applied. The analysis allowed for simultaneous independent calculation of the thickness of the thin films deposited on both sides of the glass substrate. Figure 6 shows the characteristics of the calculated thickness as a function of the distance *X* from the target axis.

The thickness calculated for the film on the front side (Figure 6, red line) is quite typical and reveals features of the theoretical cosine distribution, despite some decrease at 40–45 mm from the axis of the target. This may be an effect of the distribution of the magnetic field of the magnetron source. The front-side film had a thickness of 650–850 nm in the on-axis geometry region and of 400–600 nm in the off-axis geometry up to 100 mm away from the target axis. The film on the back side had a fairly uniform thickness in the range of 120–150 nm over the whole sample. This indicates that the film was probably formed by thermalized and backscattered particles of sputtered material.

**Figure 7:**
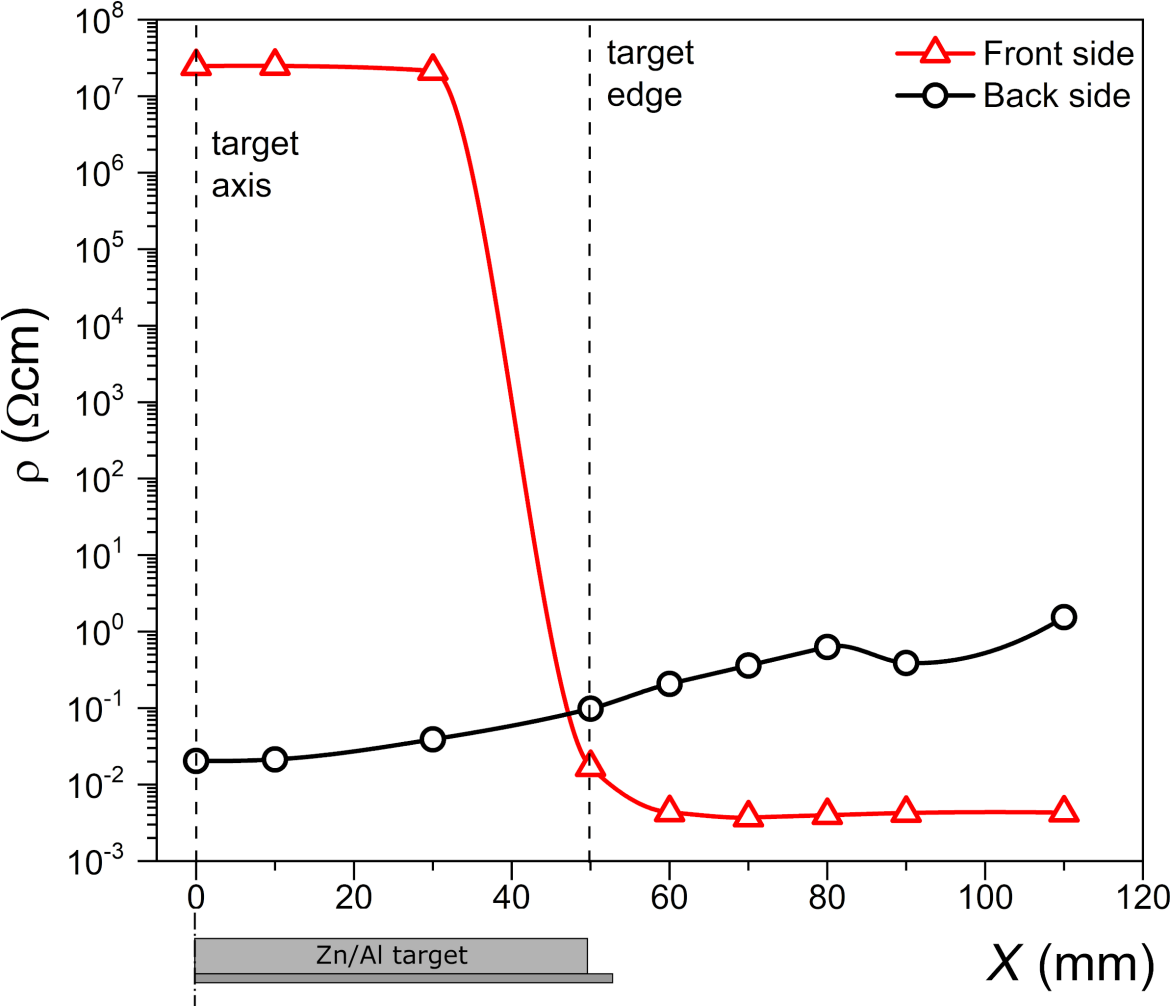
Distribution of the resistivity of thin films deposited on the front and back sides of the glass substrate as a function of the distance *X* from the Zn/Al target axis. The lines are to guide the eye.

Taking into account the thickness and the sheet resistance of the deposited films, the resistivity was calculated and plotted in Figure 7 as a function of the distance from the target axis.

The lowest resistivity was about 4 × 10^−3^ Ω·cm for the film deposited at the front side of the substrate, at a distance of about 70 mm from the target axis. Therefore, one can conclude that the area for the substrate placement with favorable conditions for the preparation of transparent and well-conductive films is located outside the radial boundary of the target.

**Figure 8:**
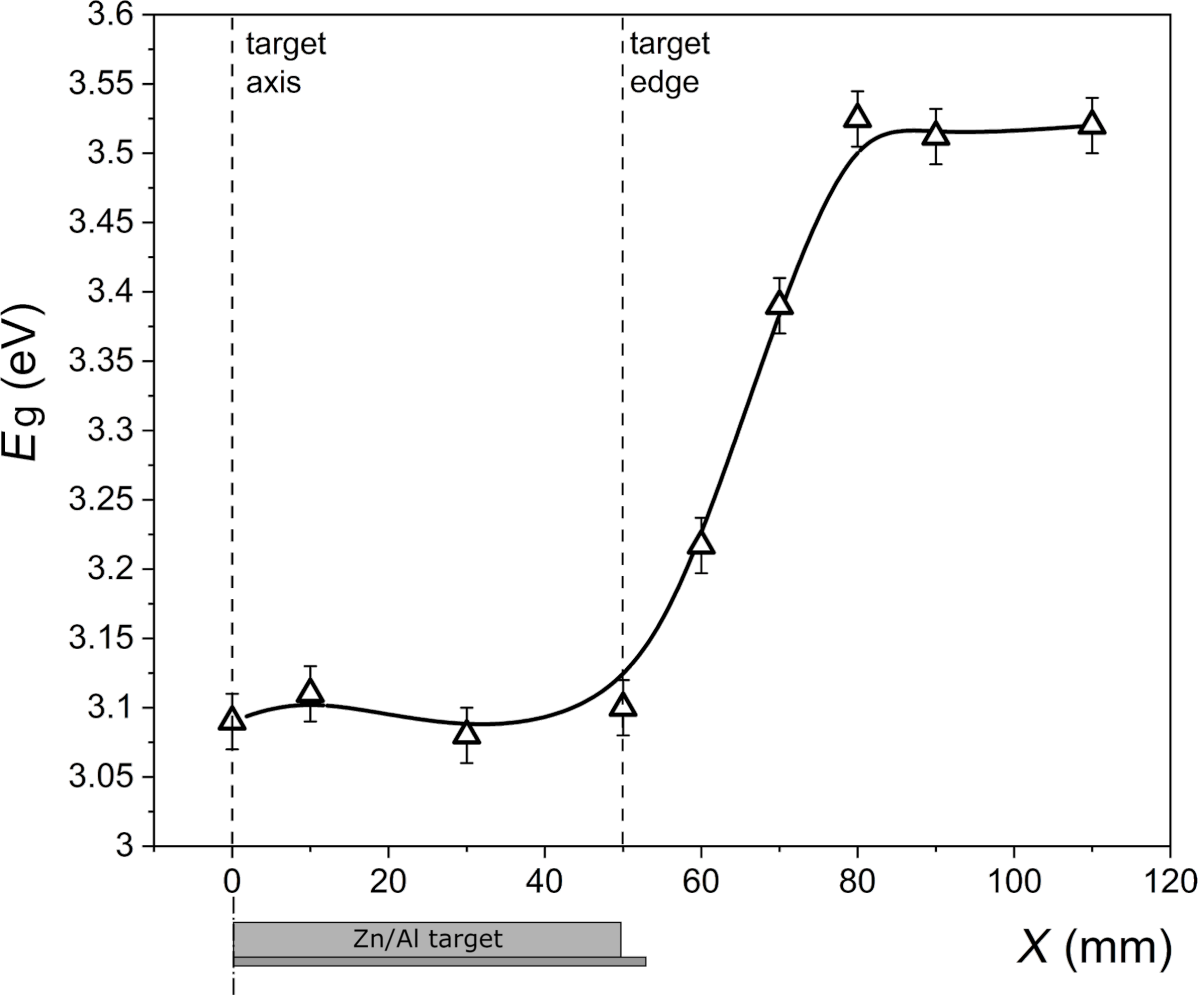
Results of the optical bandgap width estimated from optical measurements for thin films deposited at the front side of the substrate as a function of the distance *X* from the Zn/Al target axis. The line is to guide the eye.

The analysis of the optical bandgap energy (*E*_g_) as a function of *X* for the films deposited at the front side of the substrate is presented in Figure 8. As one can see, with increasing *X*, the optical bandgap increased from about 3.10 to about 3.55 eV. Such a large change suggests a relatively large change in the material composition, which will be discussed further in this paper.

**Figure 9:**
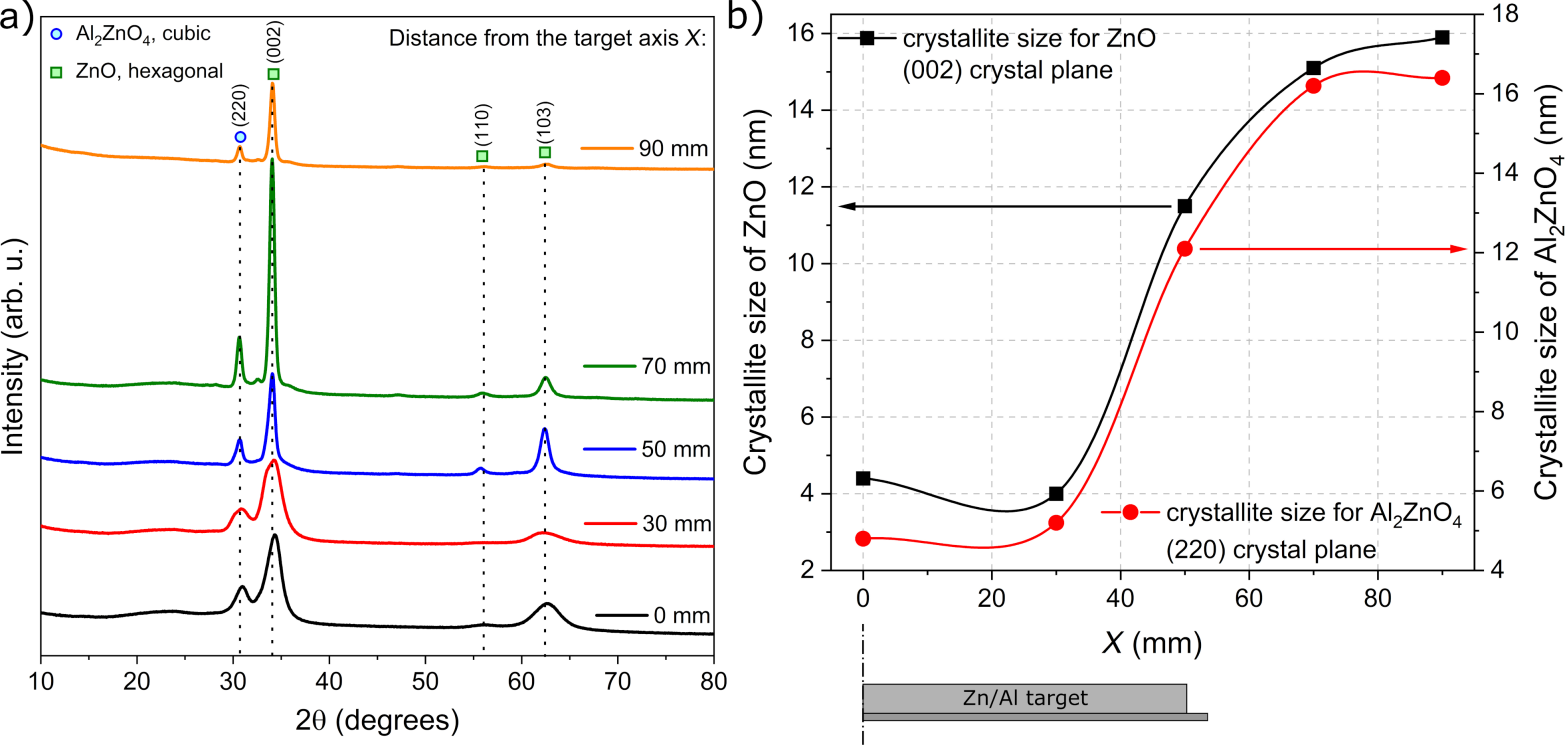
Results of the structural investigation: (a) XRD patterns and (b) crystallite sizes as functions of the distance *X* from the Zn/Al target axis. The lines in (b) are to guide the eye.

Figure 9a shows the GIXRD patterns measured for thin films deposited at different locations on the front side of the substrate. In each case, there are clearly distinguishable peaks at 2θ = 30.62° related to the (220) plane of the Al_2_ZnO_4_ cubic phase and peaks at 2θ = 34.07°, 55.97°, and 62.3° related, respectively, to the (002), (110), and (103) planes of the hexagonal ZnO phase. The crystallite size was calculated using the Scherrer formula [23]. The results of GIXRD measurements showed that all films were nanocrystalline. Thin films deposited directly above the Zn/Al target, that is, on the target axis and within a distance of up to 30 mm from the target axis, had a mixture of ZnO crystallites of the size of 4.0–4.4 nm and Al_2_ZnO_4_ crystallites of the size of 4.8–5.2 nm. With increasing distance from the target axis, the crystallite size of both phases increased considerably. Thin films placed in the area corresponding to the edge of the target, that is, at *X* = 50 mm, had crystallite sizes in the range of 11.5–12.1 nm. A further increase in *X* to 70 and 90 mm resulted in crystallite sizes of 15.9–16.4 nm. The dependence of the crystallite sizes of ZnO and Al_2_ZnO_4_ on the distance from the target axis is shown in Figure 9b.

**Figure 10:**
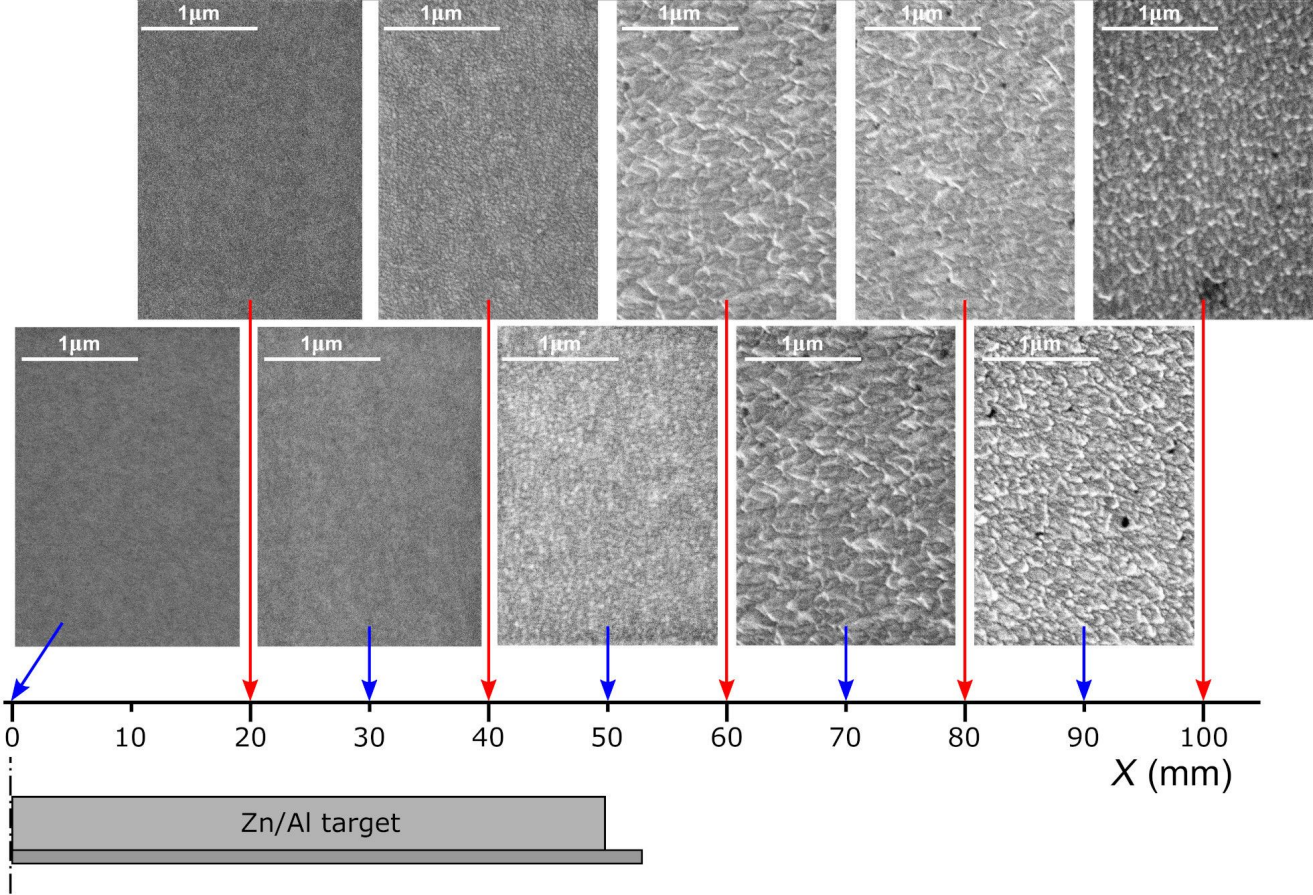
SEM images of the surface of thin films deposited on the front substrate surface at different distances *X* from the target axis.

SEM images of the surface morphology at different distances from the target axis are shown in Figure 10. All films were densely packed, homogeneous, and crack-free. For *X* ≤ 30 mm, the surface is featureless and no grains are visible. Thin films deposited directly above the target, at *X* = 40 and 50 mm, are composed of very small grains of 35–55 nm with an average size of ca. 45 nm. At larger *X*, the films had larger, densely packed, and homogeneously distributed grains of 55–80 nm with an average size of ca. 70 nm. These results agree with the XRD measurements and show that, with increasing *X*, the crystallite and grain size increases considerably.

**Figure 11:**
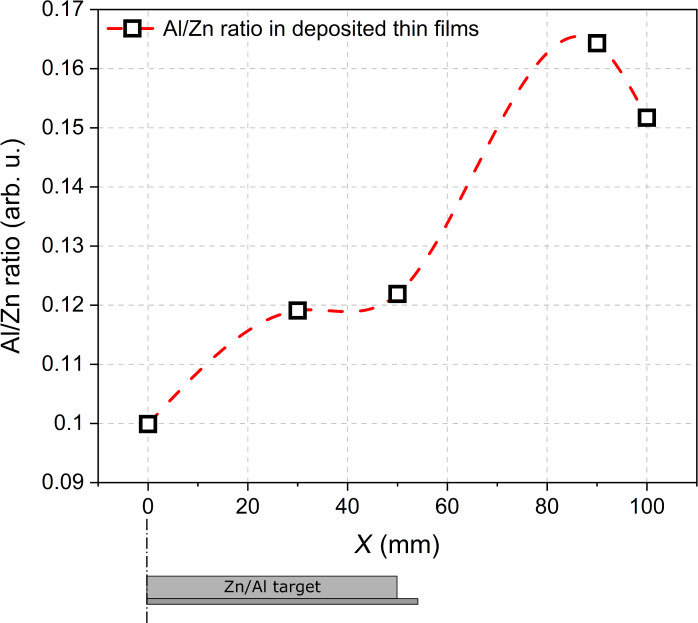
Al/Zn ratio in thin films deposited on the front side of the glass substrate as a function of the distance *X* from the Zn/Al target axis. The line is to guide the eye.

Measurements of material composition using EDS and taking into consideration only the peaks of Zn and Al revealed that with the increase of *X*, the Al/Zn ratio increased considerably from ca. 0.10 to 0.16 (Figure 11). The results presented in Figure 11 support the optical bandgap results presented in Figure 10. The higher the Al content, the wider the optical bandgap of the prepared thin film [1,24–25].

## Discussion

The analysis described in this paper showed a strong dependence of thickness as well as electrical and optical properties of the thin films depending on the position *X* of the substrate relative to the sputtered target. The thickness distribution calculated for the thin film deposited on the front side of the glass substrate is rather typical and reflects the density of the flux of species sputtered from the target and condensing at the substrate. The longer the distance from the target axis, the lower the flux density and, therefore, the lower the thickness of the condensing film.

The higher resistivity measured for films deposited on the front side of the substrate located directly above the target (on-axis geometry) probably results from the destructive effect of the bombardment of the forming Al*_x_*Zn*_y_*O films with negative oxygen ions, which was reported by many authors [11,13–15,17]. Negative oxygen ions (and also secondary electrons) directly form in the target etching zone and are then repulsed by the field near the target toward the substrate placed above it. For the case presented here, an analysis of the XRD results may be used to clarify the sudden change in the electrical properties of the deposited thin films depending on *X*. Measurements of structural properties revealed a significant increase in crystallite sizes of the ZnO and Al_2_ZnO_4_ phases, with an increase in the distance from the target axis. This increase of the crystallite size from ca. 4 to 16 nm with *X* may be caused by less energetic particles reaching the substrate related to the much longer target–substrate distance. In the case of thin films deposited off-axis, that is, outside the magnetron area, the target–substrate distance is longer than for thin films deposited directly above the magnetron (on-axis geometry). This, in turn, causes a decrease of the kinetic energy of the sputtered particles reaching the substrate and, therefore, an increase of the crystallites and grains sizes. That is also why thin films deposited directly above the target are composed of much smaller nanocrystallites of ca. 4–5 nm. Moreover, the area of the peaks related to the Al_2_ZnO_4_ and ZnO phases was calculated as the integral of the specific peaks in Figure 9a. It was found that the ratio of the area of the peaks related to the Al_2_ZnO_4_ and ZnO phases decreased significantly with increasing *X*. For thin films deposited directly above the target, the ratio of the area of the peaks, the Al_2_ZnO_4_/ZnO ratio, was approximately 0.19–0.21. For films at the edge of the target, it decreased to approximately 0.13–0.14 and remained similar with an increase of X ≥ 50 mm. Furthermore, the analysis of the material composition showed the opposite tendency; with increasing *X*, the Al content in the thin films also increased. Therefore, it can be considered that for films deposited at *X* ≥ 50 mm, more Al^3+^ ions were successfully incorporated into the host lattice as the aluminium content increases (confirmed by the EDS results) and the Al_2_ZnO_4_ phase decreases (confirmed by the XRD results). ZnO is a wide-bandgap semiconductor with high transparency in the visible wavelength range and, simultaneously, poor intrinsic conductivity. However, substitutional doping by Al replacing Zn provides an extra electron, which can populate the conduction band and lead to an increase in conductivity. This implication is in good agreement with the results of the electrical measurements as thin films deposited at *X* ≥ 50 mm are conducting, with resistivities as low as ca. 10^−3^ Ω·cm.

The results of the Al content in the deposited thin films showed that the Al/Zn ratio can even reach ca. 0.16 depending on *X*. Typically, AZO thin films consist of less than 10% aluminium [26–29] taking into account Al, Zn, and O. In this work, the material composition was measured using EDS, in which the measurement of X-ray intensities of light elements, such as oxygen, can be difficult and often subject to systematic errors. Therefore, in the case of the performed measurements, it was favorable to analyze the Al/Zn ratio because both elements are not light elements. However, it should be taken into account that AZO thin films are composed of at least 50 atom % O (ZnO/Al_2_O_3_ compound). In the case of the results here, the Al content should be at least twice as low. This would give an aluminium content of about 5–8 atom %. Moreover, in the literature regarding AZO thin films it can be found that the aluminium content can be as high as even ca. 20 atom % taking into account only Al and Zn [24–25,30–31].

Relatively well-conductive Al*_x_*Zn*_y_*O films were also obtained on the back side of the glass substrate. However, opposite to the effect observed for the front side of the substrate, as the distance from the target axis increased, the resistance of the films increased by about two orders (Figure 7). The minimum value of this resistance was about an order of magnitude greater than the minimum resistance of the layers deposited at the front of the substrate. In our opinion, film deposition on the back substrate surface was possible because the sputtered particles reached the back substrate side from collisions. They condensed on the substrate surface, which was not directly exposed to the stream of particles emitted from the sputtered target. The mean free path in an atmosphere of 2–3 × 10^−3^ mbar is of the order of millimeters, which meant that dozens of molecules collided with each other before condensing on the back side of the substrate. Clearly, only a limited number of particles reach the back surface (some condense on the front substrate surface, some on the inside of the deposition chamber), which leads to the observed lower thickness of the deposited film (Figure 6). Because of the reduced effect of the destructive bombardment with oxygen ions on the particles condensing on the back side of the substrate, favorable conditions were also created for the deposition of conductive and transparent Al*_x_*Zn*_y_*O thin films.

## Conclusion

Al*_x_*Zn*_y_*O films deposited by reactive pulsed magnetron sputtering on the front side of the glass substrates directly above (on-axis geometry) and outside the target surface (off-axis geometry) area have different electrical and optical properties testified by about nine orders difference of resistivity and about 0.4 eV difference of the optical bandgap energy. Thin films with minimum resistance were obtained on the front surface of the substrates, outside the zone directly above the target (off-axis geometry).

Analysis of structural properties showed that all thin films were nanocrystalline composites of ZnO and Al_2_ZnO_4_. Increasing *X* caused an increase of the crystallite size from ca. 4 to 16 nm, a decrease of the Al_2_ZnO_4_/ZnO ratio, and an increase in Al content. This had a direct influence on the optoelectronic properties of the deposited thin films. The best TCO film (with the lowest resistivity of 4 × 10^−3^ Ω·cm and the highest transparency of 70%) was found for samples deposited at *X* = 70–80 mm.
